# Current preventative and health promotional care offered to patients by chiropractors in the United Kingdom: a survey

**DOI:** 10.1186/s12998-015-0053-z

**Published:** 2015-03-09

**Authors:** Patricia E Fikar, Kent A Edlund, Dave Newell

**Affiliations:** Private Practice, Vienna, Austria; AECC-Anglo-European College of Chiropractic, 13-15 Parkwood Road, Bournemouth, Dorset BH5 2DF UK

**Keywords:** Chiropractic, Health promotion, Health prevention, Wellness, Non-communicable disease, Goal-setting

## Abstract

**Background:**

With increasing morbidity and mortality attributable to non-communicable disease, primary healthcare providers are urged to increasingly support people in making healthy lifestyle choices. Many chronic physical diseases associated with lifestyle behaviours have been linked to neuromusculoskeletal disorders and pain. Chiropractors, as primary healthcare professionals, are in a position to provide preventative and promotional healthcare to patients, however, it is unknown to what extent such care is provided, particularly in the United Kingdom (UK).

**Method:**

This study was a cross sectional online questionnaire distributed to four UK chiropractic associations. The responses were collected over a period of two months from March 26th 2012 to May 25th 2012. Descriptive analyses were performed to identify the trends in current practice of chiropractors in the UK. Additionally, subgroup analyses of all items were performed using Pearson Chi-Square tests to determine statistically significant differences between respondents based on gender, years in practice, educational institution and association membership.

**Results:**

Of the 2,448 members in the four participating associations, 509 chiropractors (approximately 21%) completed the survey. The great majority of UK chiropractors surveyed report evaluating and monitoring patients in regards to posture (97.1%), inactivity/overactivity (90.8%) and movement patterns (88.6%). Slightly fewer provide this type of care for psychosocial stress (82.3%), nutrition (74.1%) and disturbed sleep (72.9%). Still fewer do so for smoking (60.7%) and over-consumption of alcohol (56.4%). Verbal advice given by the chiropractor was reported as the most successful resource to encourage positive lifestyle changes as reported by 68.8% of respondents. Goal-setting is utilised by 70.7% to 80.4% of respondents concerning physical fitness issues. For all other lifestyle issues, goal-setting is used by approximately two-fifths (41.7%) or less. For smoking and over-consumption of alcohol, a mere one-fifth (20.0% and 20.6% respectively) of the responding chiropractors set goals.

**Conclusions:**

UK chiropractors are participating in promoting positive lifestyle changes in areas common to preventative healthcare and health promotion areas; however, more can be done, particularly in the areas of smoking and over-consumption of alcohol. In addition, goal-setting to support patient-provider relationships should be more widespread, potentially increasing the utility of such valuable advice and resources.

## Background

One of the greatest challenges facing global public health is the burden of non-communicable diseases (NCDs). NCDs are non-infectious and non-transmittable which include chronic, slow progressing diseases. Statistics show that in 2008, of the 57 million deaths globally, 36 million where due to NCDs of which 80% were preventable [1]. Top on the list are cardiovascular disease (most especially coronary heart disease and stroke), cancer, chronic respiratory disease and diabetes. Additionally, many countries report mental health issues as contributors to the burden by increasing the instance of the other NCDs [2]. Consequently, the World Health Organization (WHO), in its Global Strategy on Diet, Physical Activity and Health [3], is encouraging individuals to make appropriate lifestyle changes and is looking to healthcare professionals of all disciplines to be facilitators in this task on both an individual patient basis and for society as a whole. It emphasised that primary care providers have an important duty to make routine inquiries regarding these issues, helping individuals where possible to develop sustained behavioural change.

In 2006, the Council on Chiropractic Education (CCE) in the United States of America (USA) suggested a set of standards indicating specific health promotional efforts that every chiropractor should perform and health promotion and prevention methods that ideally should be part of the curriculum at accredited USA chiropractic colleges [4]. According to this standard, accepted principles of health promotion include assessing the patients’ health status, screening for risky lifestyle behaviours, and using multiple health outcome instruments. Preventative care includes educating patients regarding the impact of lifestyle on health, providing appropriate recommendations and counselling, and providing the necessary resources to promote health and wellness. For the purpose of this paper, health promotion and preventative care will take these two definitions.

Acknowledging the debate within the chiropractic profession as to whether chiropractors serve as primary care providers or spine specialists, Evans and Rupert [5] argue that health promotion and prevention are important to all chiropractors regardless of their view on their role in healthcare. They state that since many chiropractic patients seek care as part of a health maintenance programme, chiropractors are well-positioned to make routine inquiry and initiate preventative care and health promotion. In a study by Von Korff et al. [6], the authors concluded that chronic spinal pain may be co morbid with another conditions including stroke, hypertension, asthma, COPD, irritable bowel syndrome, ulcers, HIV/AIDS, epilepsy and vision problems, which are by association relevant to chiropractors. In a systematic review by Goldberg et al. [7], it was found that smoking is likely to be associated with the incidence and prevalence of non-specific back pain. Additionally, in a multiphase cross-sectional survey of musculoskeletal pain in the United Kingdom (UK) general population, Webb et al. [8] reported obesity as an important independent predictor of back pain and its severity. Furthermore, Fishbain et al. [9] found depression to be more common in chronic pain patients than in healthy controls as a consequence of the presence of chronic pain, further highlighting the importance of addressing these issues in all types of chiropractic practices.

Although some patients seek chiropractic care for pain relief and end treatment when they become asymptomatic, Evans and Rupert [5] suggest that if a patient indicates willingness to attempt to change any unhealthy behaviour, chiropractors should not simply stress personal empowerment, but be able to provide specific information and resources. UK chiropractic education not only consists of chiropractic technique and diagnosis, but provides chiropractors with a broad base in health knowledge including nutrition, physical fitness and psychosocial considerations in patient care [10]. Additionally, UK chiropractors can further their knowledge in these areas by completing continuing professional development hours through self-directed study, continuing post-graduate education or by attending national and international seminars [11].

Preventative health care and health promotion are of emerging importance to the chiropractic profession. Previous work in this area has been done by Evans et al. [12] who focused on the patients’ perspective of health promotional advice in a chiropractic teaching clinic in the USA, however, the chiropractors’ perspective on providing this type of care to their patients is unknown. Therefore, the purpose of this UK-based study is two-fold. Firstly, to determine if care is being provided by UK chiropractors in regards to preventative healthcare and health promotion, particularly in the areas of nutrition, physical fitness and exercise, psychosocial well-being, smoking and alcohol consumption. Secondly, to identify to what extent such care is provided.

## Methods

The survey questionnaire was generated from November, 2011 to March, 2012. The items were organised into categories, namely, nutrition, physical activity, psychosocial well-being, smoking and alcohol consumption. Additionally, every item on the questionnaire had a comment box where the participants could leave additional issues that did not appear on the item lists. The information submitted in these comment boxes were not included in the data analysis, but rather used for interpretation and to direct future research.

To reach a large number of chiropractors throughout the UK, the survey was distributed electronically through four UK chiropractic associations. The four participating UK associations were the British Chiropractic Association (BCA) [13], the McTimoney Chiropractic Association (MCA) [14], the Scottish Chiropractic Association (SCA) [15], and the United Chiropractic Association (UCA) [16]. The survey items were entered into the open source online survey application Limesurvey® [17]. An email bulletin with a link to the survey requesting members to participate was sent to the chiropractors by the associations’ secretaries. The survey ran once for a two-month period from the 26th of March, 2012 to the 25th of May, 2012. A reminder bulletin was sent at the end of April 2012 to all members regardless if they had already completed the survey or not, however, the bulletin indicated that they should only complete the survey once. Descriptive analyses were performed to identify the trends in current practice of chiropractors in the UK. Additionally, with the use of Statistical Package for the Social Sciences (SPSS 21.0), subgroup analyses of all items were performed using Pearson Chi-Square tests to determine statistically significant differences between respondents based on gender, years in practice, educational institution and association membership.

### Ethics

This research was ethically approved by the Anglo-European College of Chiropractic undergraduate ethics panel in March 2012.

## Results

### Cohort characteristics

Five hundred and nine participants returned fully completed surveys. The total number of members of the four participating associations, which was provided through email request by the associations’ secretaries, sums up to 2,448 member chiropractors. The 509 respondents represent approximately 21% of chiropractic membership in these four associations; however, as individual chiropractors can be members of multiple associations, the response rate is potentially higher than 21%. Furthermore, the associations’ secretaries where unable to determine the number of members on their mailing lists, so the exact number of bulletin recipients as well as the actual response rate is unknown. Of the total respondents, 63% were BCA members, with another 25% representing the UCA, 11% representing the MCA, 3% representing the SCA and 4% representing other associations. Table 1 shows the total number of members of each association at the time the study was conducted and the proportion of chiropractors in those associations who completed the survey.

**Table 1:** **Association membership and survey response rate**

**Association**	**Members** ^**1**^	**Respondents**	**Survey** **respondents** ^**2**^ **(%)**	**Membership (%)**
BCA	1352	319	63	24
MCA	544	55	11	10
SCA	145	15	3	10
UCA	407	127	25	31
Other*	unknown	19	4	N/A
**Total**	2448	516	106	21

^1^The number of members in each association was obtained through email request and the figures shown were given directly by each association.

^2^Multiplicity of membership included.

*Other associations indicated: American Chiropractic Association, Associazione Italiana Chiropratici, China Hong Kong Macao Chiropractic’ Association, Chiropractic Association of South Africa, Chiropractors’ Association of Australia, College of Chiropractors, Danish Chiropractors Association, European Chiropractors’ Union, International Chiropractic Pediatric Association, International Chiropractors Association, SOTO Europe, Swedish Chiropractic Association.

Just under half of all respondents (44.6%) were female. Most (52.1%) had practiced for 10 years or less and the majority (54.2%) were trained at the AECC. Table 2 illustrates the demographic and professional characteristics of the entire cohort.

**Table 2:** **Demographic and professional characteristics of whole cohort (N= 509)**

**Variable**	**Proportion (%)**	**N**
Gender (F)	44.6	227
Years in practice		
0-5	27.1	138
6-10	25.0	127
11-15	14.5	74
16-20	11.6	59
>20	21.8	111
Chiropractic education		
AECC	54.2	276
McTimoney	12.2	62
WIOC	19.4	99
Other*	14.2	72

*Other countries indicated for chiropractic education: Australia, Canada, New Zealand, South Africa, Sweden, UK, USA.

**Figure 1:**
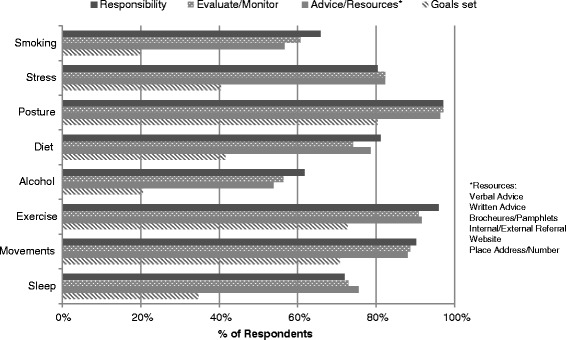
**Proportion of respondents who perceived responsibility to discuss lifestyle issues and proportion who evaluate/monitor, give advice/resources or set goals for lifestyle issues in practice.**

Of all respondents, 89.0% consider themselves to be evidence informed practitioners. Depending on the issue, 61.7 to 97.1% of the respondents agreed that the items associated with lifestyle issues were their responsibility to discuss. These issues included decreasing alcohol consumption, decreasing psychosocial stress, regular exercise, improving eating habits, improving posture, improving movement patterns, normalising sleep patterns and smoking cessation. The specific values for each issue are shown in Table 3. Similar proportions of chiropractors indicated they would evaluate/monitor (56.4-97.1%) or give advice/resources (53.8-96.3%), again depending on the specific issues listed. However, respondents’ perceived responsibility to discuss lifestyle issues with patients and the actual proportion that evaluate/monitor, give advice/resources or set goals in practice differed across all categories. These differences are visually highlighted in Figure 1. In this survey, the highest number (70.7-80.4%) of chiropractors who incorporate goal-setting and re-evaluation of goals did so in regards to physical activity. However, in all of the other areas, up to approximately two-fifths (20.0-41.7%) of the chiropractors set goals and re-evaluate. For over-consumption of alcohol and smoking, merely one-fifth (20.0% and 20.6% respectively) provide this service. Additionally, fewer chiropractors indicated they “set goals” or “re-evaluated goals”, with less than half (41.6%) setting/re-evaluating goals at the majority of visits. When asked which resource the respondents think is the most successful to prevent illness and promote their patients’ health, the majority (68.8%) indicated verbal advice, followed in descending order by written advice (10.6%), referral to another health professional (internal or external) (6.3%), brochures and pamphlets (3.3%), internet website address (1.4%) and a place address or contact number (0.2%). Table 3 summarises the respondents’ practice characteristics and behaviours.

**Table 3:** **Practice characteristics and behaviours of whole cohort (N= 509)**

**Question**	**Proportion (%)**	**N**
**Evidence informed?**		
Yes	89.0	453
No	2.4	12
No answer	8.6	44
**Lifestyle issues considered responsible to discuss**		
Decrease alcohol consumption	61.7	314
Decrease psychosocial stress	80.4	409
Regular exercise	95.9	488
Improve eating	81.1	413
Improve posture	97.1	494
Improve movement patterns	90.2	459
Normalise sleep patterns	71.9	366
Smoking cessation	65.8	335
Other	11.8	60
**Behaviours Evaluated/monitored**		
Over-consumption alcohol	56.4	787
Psychosocial stress	82.3	419
Inactivity/Over activity	90.8	462
Poor diet	74.1	377
Poor posture	97.1	494
Faulty movement patterns	88.6	451
Disturbed sleep	72.9	371
Smoking	60.7	309
Other	7.7	39
**Advice/resources given**		
Over-consumption alcohol	53.8	274
Psychosocial stress	82.3	419
Inactivity/Over activity	91.6	466
Poor diet	78.6	400
Poor posture	96.3	490
Faulty movement patterns	88.0	448
Disturbed sleep	75.4	384
Smoking	56.6	288
Other	8.8	45
**Set goals/re-evaluate progress**		
Over-consumption alcohol	20.6	105
Psychosocial stress	40.5	206
Inactivity/Over activity	72.7	370
Poor diet	41.7	212
Poor posture	80.4	409
Faulty movement patterns	70.7	360
Disturbed sleep	34.8	177
Smoking	20.0	102
Other	6.7	34
**Frequency of setting/review goals**		
Up to 50% of visits	48.5	247
51-100% of visits	41.6	212
Never	4.7	24
Only per patient request	5.1	26
**Successful resources to prevent disease/promote health**		
Verbal advice from chiropractor	68.8	350
Written advice from chiropractor	10.6	54
Brochures/Pamphlets	3.3	17
Internal/External referral	6.3	32
Internet website address	1.4	7
Place address/contact number	0.2	1
No answer	9.4	48

### Subgroup analysis

In order to determine any differences between subgroups, analyses were carried out using Pearson Chi-Square tests. This revealed that there was a statistically significant greater proportion of males with >20 years in practice, χ^2^ (4, N =489) =13.85, p = .008) reflecting an increasing female representation in the profession over the most recent decades. Additionally, in regards to years in practice and association membership, the UCA had a statistically significant over-representation of respondents with fewer years in practice, χ^2^ (4, N =489) =17.18, p = .002), while the BCA was over-represented by respondents with greater years in practice, χ^2^ (4, N =489) =16.66, p = .002). This was not seen in the MCA or SCA.

In relation to patient behaviours, a significantly greater number of female respondents reported disturbed sleep, χ^2^ (1, N =489) =9.74, p = .002), and psychosocial stress, χ^2^ (1, N =489) =6.99, p = .008), as being behaviours they evaluate and/or monitor. Conversely, males reported poor posture, χ^2^ (1, N =489) =5.98, p = .014), with higher frequency than females. In regards to the lifestyle changes for which the respondents set and/or re-evaluate goals with their patients, respondents in practice for ≤20 years reported doing so more frequently in regards to poor posture, χ^2^ (4, N =489) =12.97, p = .011). On the other hand, respondents in practice for >20 years reported with higher frequency goal setting/re-evaluation for smoking, χ^2^ (4, N =489) =11.35, p = .023).

## Discussion

This study indicates that the surveyed UK chiropractors are participating in preventative healthcare and health promotion activities, encouraging patients to make positive lifestyle changes especially in regards to physical activity and fitness. Although less in comparison to physical activity, our findings indicate that a fair degree of nutritional and psychosocial care is provided. These are potentially important components not only to individual patients, but to public health initiatives as highlighted by O’Connor et al. [18]. The authors suggest that chiropractors, given the practicalities of their work, have the opportunity to be opportunistic screeners and referrers for a variety of conditions that can potentially decrease mortality and morbidity at minimal cost, an important consideration emerging across the global health care sector.

The results also show that just over half of the chiropractors reported smoking and over-consumption of alcohol as their responsibility to discuss. However, less of these same clinicians evaluate and monitor their patients for these behaviours. This is surprising as over-consumption of alcohol and smoking are not only known to lead directly to the development of NCDs, but additionally have quite severe consequences on skeletal and nervous system health [19-21]. Nevertheless, the proportion of UK chiropractors reporting that they provide advice for smoking cessation is similar to the amount of advice being offered by other healthcare professionals to patients in the USA [22,23]. In the UK, Coleman et al. [24] states that many opportunities to discuss smoking with patients are not being utilised by general practitioners in order to avoid negative responses from patients and many practitioners do not raise the topic unless the patients present with smoking-related problems. It appears to be an area of care that needs more attention from healthcare professionals as a whole and chiropractors could have a positive role to play.

As preventative care and health promotion focus on the daily choices an individual makes regarding their long-term health objectives, goal-setting should be a core component. In a study by Holliday et al. [25], neurological rehabilitation patients in the UK who were encouraged to participate in goal-setting found such goals to be more relevant than those set primarily by the providers as they felt a greater sense of autonomy and satisfaction when they were part of the process. Goal-setting where patients and providers co-create health by focusing on a specific problem, setting realistic objectives, and developing action plans should be viewed as a core service according to Von Korff et al. [26]. In our study, other than for physical fitness issues, goal-setting appears to be under-utilised with only up to two-fifths (41.7%) of the chiropractors setting and re-evaluating goals depending on the specific lifestyle issue and only one-fifth doing so for issues of over-consumption of alcohol (20.0%) and smoking (20.6%). A case therefore could be made to encourage more chiropractors to adopt goal-setting in key areas of preventative healthcare and health promotion.

### Study limitations

A significant limitation of this study is the estimated 79% non-response rate from the UK chiropractors in this survey. Although the approximate 21% response rate allows us to draw conclusions regarding the practices of those who responded, the UK chiropractic profession is not represented in its entirety. This leads to less than robust confidence in the generalisability of these attitudes and practices to the wider profession.

Additionally, the chiropractors were asked to report general tendencies, not specific patient encounters, and interpretation between chiropractors may differ. This is particularly important where clinicians were asked to report the number of patient visits where goal-setting and re-evaluation of goals took place. Additionally, false reports, conscious or subconscious, might have occurred; however, this is an unavoidable limitation of the study design.

Lastly, some issues were not included in the survey, but may have an impact on decreasing NCDs as suggested by the respondents in the comment boxes. These included pregnancy and birth plans, regular weight checks, work-life balance, supplements, prescription drugs, vaccinations, ergonomics, exposing oneself to adequate sunlight and spiritual pursuits.

### Future research

This survey explored the degree with which UK chiropractors engage with patients concerning efforts to support the potential reduction in the burden of NCDs. While being only a snapshot of the current situation for the chiropractors who responded, this study could serve as a starting point for further work in this area in the UK. Future studies in this field will not only be of interest to patients and chiropractors, but potentially to other health care professionals as well as policy makers. Further exploration of the patients’ perspective and the reasons why chiropractors do or do not consider it their responsibility to incorporate such care into their practices as well as seeking insight into successful methods being utilised in chiropractic practices should be explored.

## Conclusion

Links have been shown between chronic disease, smoking, obesity and depression with pain, particularly spinal pain. With the WHO calling on primary healthcare professionals to assist patients in developing sustained lifestyle changes, the goal of this study was to explore the chiropractic professions’ engagement with preventative healthcare and health promotion. This survey of a subset of UK chiropractors shows that a good degree of preventative healthcare and health promotion is already provided, especially in regards to physical activity, nutrition and psychosocial stress. However, goal-setting and re-evaluation of goals could be incorporated more routinely by UK chiropractors into their practices. The chiropractic profession has a valuable role in preventative healthcare and health promotion and many of those surveyed are already participating in decreasing the burden of NCDs in various aspects of patient care. However, as in most things, there is considerable room for improvement if desired.

## Abbreviations

BCA: British Chiropractic Association
CAM: Complementary and alternative medicine
MCA: McTimoney Chiropractic Association
NCD: Non-communicable disease
SCA: Scottish Chiropractic Association
SPSS: Statistical Package for the Social Sciences
UCA: United Chiropractic Association
UK: United Kingdom
USA: United States of America
WHO: World Health Organization
